# Quantum Iterative Reconstruction for Low-Dose Ultra-High-Resolution Photon-Counting Detector CT of the Lung

**DOI:** 10.3390/diagnostics12020522

**Published:** 2022-02-18

**Authors:** Thomas Sartoretti, Damien Racine, Victor Mergen, Lisa Jungblut, Pascal Monnin, Thomas G. Flohr, Katharina Martini, Thomas Frauenfelder, Hatem Alkadhi, André Euler

**Affiliations:** 1 Institute of Diagnostic and Interventional Radiology, University Hospital Zurich, University of Zurich, CH-8091 Zurich, Switzerland; thomas.sartoretti@usz.ch (T.S.); victor.mergen@usz.ch (V.M.); lisa.jungblut@usz.ch (L.J.); katharina.martini@usz.ch (K.M.); thomas.frauenfelder@usz.ch (T.F.); hatem.alkadhi@usz.ch (H.A.); 2 Institute of Radiation Physics (IRA), Lausanne University Hospital (CHUV), University of Lausanne (UNIL), CH-1010 Lausanne, Switzerland; damien.racine@chuv.ch (D.R.); pascal.monnin@chuv.ch (P.M.); 3 Siemens Healthcare GmbH, 91052 Forchheim, Germany; thomas.flohr@siemens-healthineers.com

**Keywords:** phantoms, imaging, tomography, X-ray computed, lung

## Abstract

The aim of this study was to characterize image quality and to determine the optimal strength levels of a novel iterative reconstruction algorithm (quantum iterative reconstruction, QIR) for low-dose, ultra-high-resolution (UHR) photon-counting detector CT (PCD-CT) of the lung. Images were acquired on a clinical dual-source PCD-CT in the UHR mode and reconstructed with a sharp lung reconstruction kernel at different strength levels of QIR (QIR-1 to QIR-4) and without QIR (QIR-off). Noise power spectrum (NPS) and target transfer function (TTF) were analyzed in a cylindrical phantom. 52 consecutive patients referred for low-dose UHR chest PCD-CT were included (CTDI_vol_: 1 ± 0.6 mGy). Quantitative image quality analysis was performed computationally which included the calculation of the global noise index (GNI) and the global signal-to-noise ratio index (GSNRI). The mean attenuation of the lung parenchyma was measured. Two readers graded images qualitatively in terms of overall image quality, image sharpness, and subjective image noise using 5-point Likert scales. In the phantom, an increase in the QIR level slightly decreased spatial resolution and considerably decreased noise amplitude without affecting the frequency content. In patients, GNI decreased from QIR-off (202 ± 34 HU) to QIR-4 (106 ± 18 HU) (*p* < 0.001) by 48%. GSNRI increased from QIR-off (4.4 ± 0.8) to QIR-4 (8.2 ± 1.6) (*p* < 0.001) by 87%. Attenuation of lung parenchyma was highly comparable among reconstructions (QIR-off: −849 ± 53 HU to QIR-4: −853 ± 52 HU, *p* < 0.001). Subjective noise was best in QIR-4 (*p* < 0.001), while QIR-3 was best for sharpness and overall image quality (*p* < 0.001). Thus, our phantom and patient study indicates that QIR-3 provides the optimal iterative reconstruction level for low-dose, UHR PCD-CT of the lungs.

## 1. Introduction

Photon-counting detector computed tomography (PCD-CT) is an emerging technology that enables the direct conversion of incident x-ray photons into an electrical signal. As compared to conventional energy-integrating detector (EID)-CT systems, PCD-CT has shown improved spatial resolution, lower image noise, and higher contrast-to-noise ratio (CNR) [1,2,3,4,5,6,7,8,9]. Previous studies on preclinical prototype PCD-CT suggest that these benefits can be translated to an improved diagnosis of pulmonary nodules [10] and to improvements in shape and texture image information due to the higher spatial resolution of PCD-CT as compared to EID-CT [11,12].

Recently, the first whole-body full field-of-view dual-source PCD-CT [13,14,15] has become available for clinical use. This system overcomes the limitations of previous PCD-CT prototype systems by offering a 50 cm scan FOV and 5.76 cm longitudinal detector coverage with automatic exposure control in both angular and longitudinal directions [14]. The PCD of the system offers a 0.15 × 0.176 mm^2^ detector element size (projected to the iso-center) that enables ultra-high spatial resolution (UHR) without the radiation dose penalty of other conventional CT systems [14]. Specifically, this new UHR-mode surpasses prior UHR implementations on EID-CT systems in terms of spatial resolution and it does not need a comb filter, which was traditionally associated with a decreased dose efficiency of 50% [16]. The detector pixels of the PCD can either be read out independently in the UHR data acquisition mode or 2 × 2 pixels can be binned to a “macropixel” in the “standard” data acquisition mode (z-coverage, 57.6 mm; 144 × 0.4 mm at the isocenter). The z-coverage in the UHR data acquisition mode is limited to 24 mm (120 × 0.2 mm at the isocenter) [17]. In the context of lung imaging, the use of UHR imaging is of particular clinical interest, as fine parenchymal changes and disorders can be visualized in high quality at low noise levels [18,19].

While hardware improvements are critical to ensure high-quality CT, image reconstruction techniques also contribute decisively toward image quality and perception [20,21]. Latest generation iterative reconstruction (IR) algorithms considerably reduce image noise thus becoming an important strategy to achieve diagnostic image quality for lung CTs at low-radiation doses [22,23,24].

For PCD-CT, conventional IR algorithms as developed for EID-CT systems are not suitable due to a variety of technical factors such as the increased data complexity, spectral information, and noise model [20]. Therefore, a new IR algorithm named quantum iterative reconstruction (QIR) has been introduced. The QIR algorithm has four strength levels and it is tailored toward the hardware and software requirements of the PCD-CT system. Importantly, QIR can be combined with all acquisition modes of the PCD-CT, including the UHR mode. Given these considerations, we hypothesize that a combination of QIR and UHR may be particularly interesting for lung imaging as high-quality, ultra-high-resolution imaging at very low radiation doses may be achieved.

The purpose of our study was to characterize the image quality and to determine the optimal strength level of QIR for low-dose, ultra-high-resolution, photon-counting detector CT of the lung using various quantitative and qualitative metrics of image quality in a phantom and in patients.

## 2. Materials and Methods

### 2.1. Phantom

To assess noise texture and spatial resolution, the noise power spectrum (NPS) and target transfer function (TTF) were measured on images of a 25 cm diameter cylindrical water phantom. The phantom was scanned using the same acquisition and reconstruction parameters as in patients (see Section 2.2 for details below).

High-contrast TTF was calculated from central cylindrical inserts with a diameter of 10 cm made of Polytetrafluoroethylene (PTFE—mean CT number 1050 HU at 120 kVp). The method of TTF computation used in this study was previously described in detail [25]. Seventy-two radial edge spread functions (ESF) were measured every 5 degrees from 280 identical slices of each insert, using a 10-degree angular aperture. The modulus of the Fourier transforms of the radial ESFs gave 72 radial TTFs for each angular sector. Each 1D radial TTF was the average of the 72 angular TTFs.

A homogeneous volume of water on one side of the phantom without an insert was used for the NPS measurement. The NPS was measured in homogeneous slices of water as previously shown [25], based on the recommendations of the International Commission of Radiation Units and Measurements Reports 54 and 87 [26,27]. The 2D NPS were calculated from a square region of interest (ROI) of 270 × 270 pixels (158 × 158 mm^2^) centered in the middle of the phantom over 400 identical slices, yielding a statistic of over 29 million pixels. The angular mean of the 2D NPS resulted in a 1D radial NPS. In detail, the 2D in-plane NPS amplitude was expressed by a one-dimensional (1D) radial NPS. This 1D radial NPS was obtained by plotting the 2D in-plane NPS amplitude as a function of the radial frequency in polar coordinates. Thus, the 1D NPS represented the 2D in-plane NPS averaged over all angular frequencies. No correction of pixel values, such as trend removal, was performed on the images prior to the calculation of the NPS.

### 2.2. Patients

This study was approved by the institutional review board and local ethics committee. All patients provided written informed consent. Between March and August 2021, we retrospectively assessed 52 consecutive patients (30 male, 22 female; age 61 ± 13 years; body mass index (BMI) 24.3 ± 5.6 kg/m^2^; effective patient diameter defined as the square root of the product of the anteroposterior and the lateral diameter 275 ± 43 mm) who underwent low-dose UHR lung CT at our radiology department. Inclusion criterion was 18 years or older. Exclusion criteria were metal artifacts in the chest that could potentially affect image quality. The diagnoses of the patients were as follows: pulmonary nodule (n = 19), interstitial lung disease (n = 15), pneumonia (n = 8), carcinoma/metastases/sarcoma (n = 6), pulmonary embolism (n = 1), rib fractures (n = 2), and elevation of the diaphragm (n = 1).

### 2.3. Data Acquisition

Both in the phantom and in patients, images were acquired on the same first-generation clinical dual-source PCD-CT (NAEOTOM Alpha, Siemens Healthcare GmbH, Forchheim, Germany) in the single-source UHR mode. In the current system version, the UHR mode employs a single energy threshold at 20 keV [14]. The following scan parameters were used: 120 kVp, detector collimation of 120 × 0.2 mm, pitch 0.85, and gantry rotation time 0.5 s. The tube current was automatically adjusted to achieve an image quality level (IQ level) of 15 for each scan. The IQ-level represents quality reference milliampere-seconds (mAs), which denotes effective mAs applied for the protocol-specific reference water-equivalent diameter with a CT geometry correction, particularly for the effect of the focal spot to iso-center distance. Therefore, the image quality level provides a system-independent image quality definition. Radiation dose parameters of the applied protocol in the patients were as follows: effective mAs 13.1 ± 7.3, CTDI_vol_ 1 ± 0.6 mGy, dose length product (DLP) 35.5 ± 19.8 mGy*cm, and size specific dose estimate (SSDE) 1.3 ± 0.6 mGy. In the phantom, CTDI_vol_ was 0.9± 0.1 mGy.

### 2.4. Image Reconstruction

Axial images were reconstructed with QIR-off and with all strength levels of QIR (QIR 1-4). A sharp, fine-detail reconstruction kernel (Bl64), a section thickness of 1.5 mm, an increment of 1mm, and a matrix size of 512 × 512 pixels were used.

In brief, QIR is an iterative reconstruction approach which performs a statistical optimization of spectral data and corrects for geometric cone beam artifacts. The configuration QIR-off is equivalent to a weighted filtered back projection (FBP) reconstruction. The QIR strength levels 1–4 trigger an additional statistical optimization in terms of a globally reduced target noise level. Statistical optimization (noise reduction) is based on locally adaptive iterative regularization. In contrast to naive regularization in standard model-based IR, adaptive regularization incorporates statistical weighting directly in the regularization step. This corresponds to using a locally adaptive noise model in order to separate information and noise by (local) signal-to-noise analysis of the data content and partially subtract detected noise in each iteration step. In contrast to single-spectra application, all spectra need to be treated consistently for statistical improvement. This means that the structural content is assumed congruent for all spectra, which is satisfied due to spatially and temporarily consistent acquisition of the spectral data, however, with individual contrast as well as individual local noise levels. Therefore, regularization can be constrained geometrically, utilizing structural information derived from common data. Further details regarding QIR can be found elsewhere [28].

### 2.5. Quantitative Analysis

A fully automatic computational pipeline for in vivo quantitative image analysis was developed in the R programming language. Specifically, the CT patient images were first loaded into the program. Then, a segmentation of the lungs was performed using a previously validated segmentation algorithm that leverages thresholding- and region-based segmentation methodology to create lung templates [29]. Thereby all voxels of the lungs were extracted from the original dataset. Then, noise maps were computed specifically for the lungs and the global noise index (GNI) was calculated. The GNI metric was adapted from Christianson et al. [30] and represents a robust measure to quantify the noise level in vivo across the whole target imaging volume of a single examination. By using the noise maps as derived from each image, a histogram of the noise distribution over the lungs could be calculated. From this histogram, the mode value was extracted which then corresponded to the GNI.

**Figure 1:**
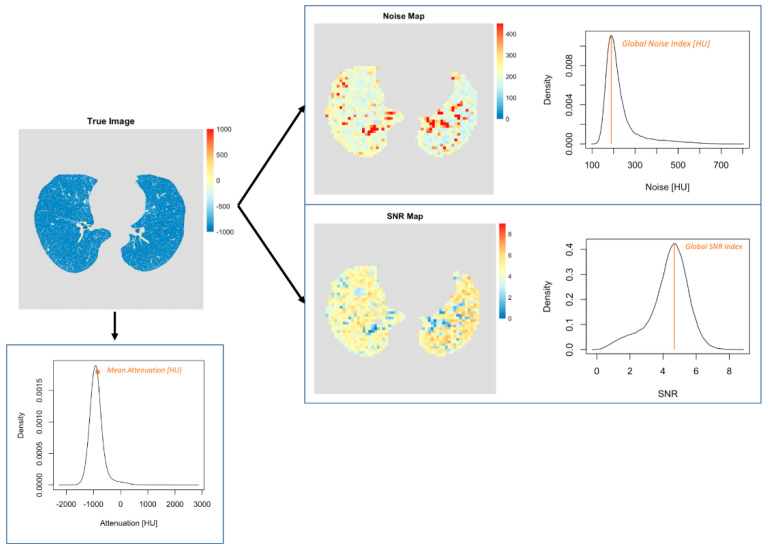
Visual representation of the quantitative image analysis in patients. First, the lungs were segmented automatically from the CT image sets. Second, mean CT attenuation was measured. Third, noise and signal-to-noise ratio maps were generated. The mode values of the corresponding distributions were defined as GNI and GSNRI.

Furthermore, SNR maps were generated for the whole lung and the global SNR index (GSNRI) was extracted. The SNR maps were computed by dividing the attenuation by the standard deviation (SD) of the attenuation (i.e., noise) within a target region. Then, a histogram of the SNR distribution over the lungs was generated and the mode value of the histogram was extracted representing the GSNRI as a global metric to quantify the overall SNR performance across the whole lung. Finally, the mean attenuation of all voxels across the lungs was recorded. A visual representation of this procedure is provided in Figure 1.

### 2.6. Qualitative Analysis

A subjective image quality assessment of patient scans was performed by two readers (V.M. and L.J., radiology residents with two and three years of experience, respectively) in a randomized, blinded fashion as recommended in a previous study [31]. The images were evaluated with 5-point Likert scales for overall image quality (1—unacceptable, 2—fair, 3—moderate, 4—good, and 5—excellent), image sharpness (1—unacceptable reduction of sharpness, 2—significantly reduced sharpness and blurring with adjacent structures, 3—minimally reduced sharpness with blurring aspect to adjacent structures, 4—minimally reduced sharpness, and 5—excellent sharpness), and image noise (1—unacceptable image noise, 2—above average noise, 3—average image noise, 4—less than average noise, and 5—minimal image noise).

### 2.7. Statistical Analysis

The data distribution was checked using histograms, boxplots, and quantile-quantile plots. Friedman tests with post-hoc Wilcoxon signed-rank tests were used to assess differences in qualitative metrics (and non-normally distributed quantitative data). The interreader agreement of qualitative scores was quantified with Krippendorff’s α coefficients (0.0–0.20 = poor agreement, 0.21–0.40 = fair agreement, 0.41–0.60 = moderate agreement, 0.61–0.80 = substantial agreement, and 0.81–1.00 = almost perfect agreement). One-way repeated measures ANOVAs with post-hoc paired t-tests were used to test for differences in normally distributed quantitative metrics. The *p*-values were corrected for multiple comparisons with the Benjamini–Hochberg procedure. Two-tailed *p*-values < 0.05 were considered statistically significant. The quantitative data is presented as mean ± standard deviation (SD) while the qualitative data is presented as median (interquartile range). All statistical analyses were performed in the R statistical software (version 4.0.2; R Foundation for Statistical Computing, Vienna, Austria, https://www.R-project.org/, last accessed on 4 December 2021).

## 3. Results

### 3.1. Phantom

**Figure 2:**
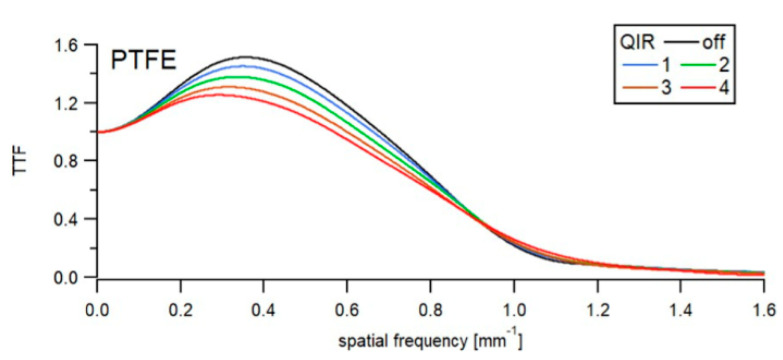
High contrast TTF as a function of image reconstruction. Graphs show TTF for a high-contrast task (PTFE—Polytetrafluoroethylene) as a function of spatial frequency among reconstructions. A slight shift towards lower frequencies was observed with increasing QIR level indicating slightly decreased spatial resolution.

The impact of QIR on high-contrast TTF is summarized in Figure 2 and Table 1. An increase in the QIR level decreased noise considerably and only decreased spatial resolution slightly. For QIR-off, the TTF peaked at 1.52. 

**Figure 3:**
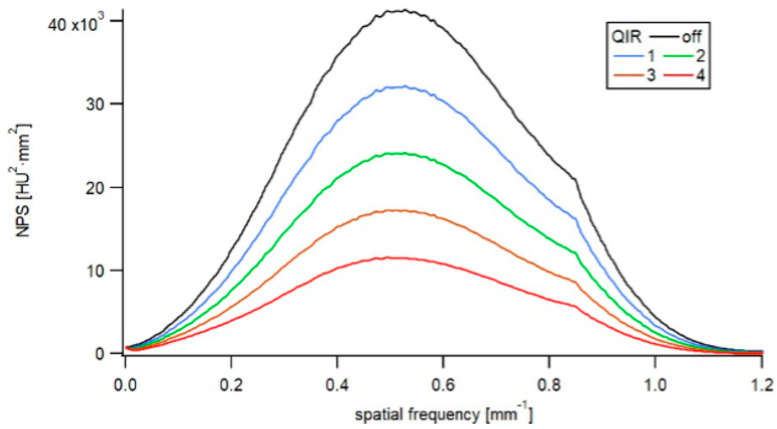
NPS as a function of image reconstruction. The NPS showed decreasing noise magnitude with increasing QIR level. The shape of the NPS indicated similar noise texture among reconstructions.

Increasing the QIR level from QIR-1 to QIR-4 reduced the noise magnitude to 78%, 59%, 43%, and 29% of the initial noise level (QIR-off), respectively. The NPS peak frequency was affected only minimally by QIR (Figure 3 and Table 2), with QIR levels 1–3 exhibiting no shift in peak frequency relative to QIR-off and QIR-4 exhibiting a shift of −6.7% in peak frequency relative to QIR-off. Therefore, changing the QIR level had no relevant effect on the NPS shape and frequency bandwidth. The noise texture of the images was not modified by the choice of the QIR level.

### 3.2. Patients

**Figure 4:**
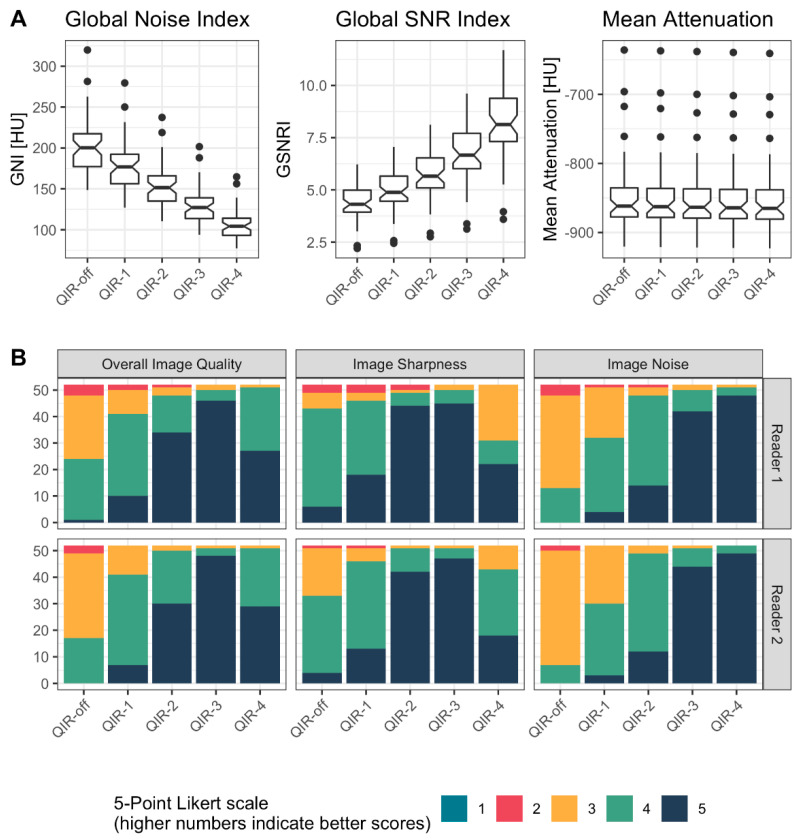
Detailed overview of the results from qualitative and quantitative analysis in patients. (**A**) shows the results from quantitative analysis by means of notched boxplots. A linear decrease of GNI and a linear increase of GSNRI were observed with increasing level of QIR. The CT attenuation was similar among reconstructions. (**B**) shows the results from qualitative analysis by means of stacked bar plots. While QIR-4 achieved highest ratings for image noise, QIR-3 performed best for sharpness and overall image quality.

**Figure 5:**
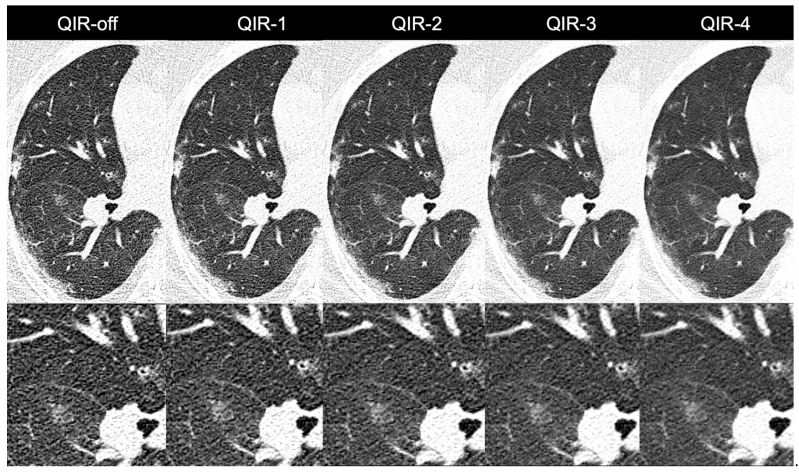
Images of a 55-year-old female patient with atypical pneumonia. Noise was reduced considerably when changing from QIR-off to higher levels of QIR. Note the slightly smoother appearance of QIR-4 as opposed to lower levels of QIR indicating slightly reduced sharpness.

**Figure 6:**
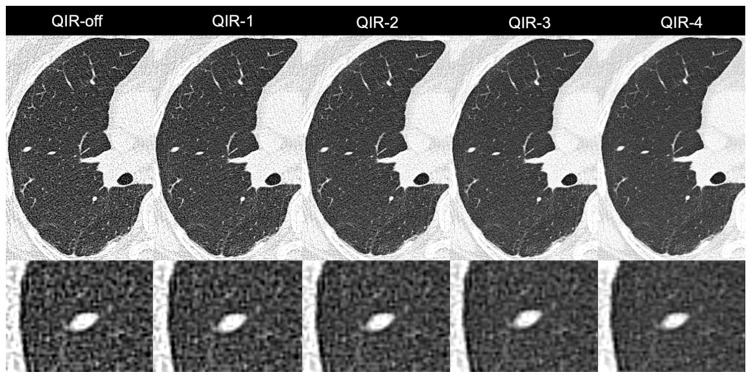
Images of a 58-year-old male patient with a solid 6 mm pulmonary nodule in the lateral middle lobe. Noise was reduced considerably when switching from QIR-off to higher levels of QIR. Note the slightly smoothed appearance of the nodule on QIR-4 as opposed to lower levels of QIR.

A detailed overview of the results is provided in Figure 4 and Table 3. Representative image examples are provided in Figure 5 and Figure 6.

#### 3.2.1. Quantitative Analysis

The GNI decreased linearly from QIR-off (202 ± 34 HU) to QIR-4 (106 ± 18 HU), with significantly lower image noise for QIR-4 compared to all other reconstructions (all, *p* < 0.001); and QIR-4 achieved a 47.5% noise reduction relative to QIR-off. The GSNRI increased linearly from QIR-off (4.4 ± 0.8) to QIR-4 (8.2 ± 1.6) with significantly higher GSNRI for QIR-4 compared to all other reconstructions (all, *p* < 0.001). Specifically, QIR-4 achieved an 86.9% higher SNR compared to QIR-off.

The attenuation values in lung parenchyma ranged from −849 ± 53 HU for QIR-off to −853 ± 52 HU for QIR-4, with a mean attenuation decreasing by an average of 1 HU per level of QIR from QIR-off to QIR-4. Still, this difference was statistically significant (all, *p* < 0.001).

#### 3.2.2. Qualitative Analysis

The interreader agreement ranged from substantial to almost perfect (α = 0.71/0.825/0.878/ for image sharpness/overall image quality/image noise, respectively). For image noise, QIR-4 (reader 1: 5; [5,5] and reader 2: 5; [5,5]) significantly outperformed all other reconstructions (*p* < 0.001—*p* = 0.02 for both readers), followed by QIR-3 (reader 1: 5; [5,5] and reader 2: 5; [5,5]). For image sharpness, QIR-2 (reader 1: 5; [5,5] and reader 2: 5; [5,5]) and QIR-3 (reader 1: 5; [5,5] and reader 2: 5; [5,5]) outperformed all other reconstructions (all, *p* < 0.001 for both readers) without significant differences between QIR-2 and QIR-3 (reader 1: *p* = 0.23, reader 2: *p* = 0.16). For overall image quality, QIR-3 (reader 1: 5; [5,5] and reader 2: 5; [5,5]) outperformed all other reconstructions (all, *p* < 0.001 for both readers).

## 4. Discussion

In this study, we assessed the image quality and optimal strength level of an iterative reconstruction algorithm (quantum iterative reconstruction (QIR)) designed for photon-counting detector CT (PCD-CT) for low-dose, ultra-high-resolution (UHR) CT of the lungs. In our phantom and patient study, we found that high levels of QIR enabled considerable noise reductions, increased SNR, and improved subjective image quality without affecting noise texture. Although higher levels of QIR were associated with slightly decreased objective spatial resolution, subjective image sharpness was deemed best on QIR-2 and QIR-3 relative to all other reconstructions. Thus, when considering all metrics, our results indicate that QIR-3 provides the optimal trade-off between objective and subjective image quality, noise reduction, spatial resolution, and noise texture.

To date, only a few studies have explored the potential of PCD-CT for lung imaging. Specifically, previous studies sought to establish the added value of PCD-CT relative to EID-CT. Bartlett et al. assessed the performance of a prototype PCD-CT system for high-resolution lung imaging [18]. Twenty-two patients underwent both PCD-CT imaging and EID-CT imaging with matching dose levels. Images were reconstructed either with wFBP or IR algorithms (ADMIRE for EID-CT and SAFIRE for PCD-CT) as well as with 512 and/or 1024 matrix sizes. The authors concluded that high-resolution PCD-CT may outperform current EID-CT systems for the visualization of higher-order bronchi and bronchial walls without compromising nodule visualization [18]. 

Jungblut et al. provided initial data on the performance of a first-generation clinical dual-source PCD-CT for lung nodule imaging [17]. An anthropomorphic chest-phantom was imaged both on the PCD-CT and on an EID-CT system at various matching dose levels. Importantly for PCD-CT, images were reconstructed with QIR strength level 3. The authors concluded that PCD-CT provides superior image quality to dose-matched EID-CT across a range of dose levels [17]. 

To the best of our knowledge, the phantom study by Jungblut et al. was the first to introduce a clinical PCD-CT system for lung imaging and more specifically QIR as a novel IR algorithm optimized for PCD-CT imaging [17]. In prior studies, such as that of Bartlett et al. [18], images were acquired with prototype PCD-CT systems thereby relying on wFBP or conventional EID-CT-based IR algorithms (such as SAFIRE) for image reconstruction. 

Since its first introduction in 2009, several generations of IRs have been developed for EID-CT thus enabling substantial radiation dose and/or image noise reductions as compared to FBP [20,21,24]. Current IR algorithms are, however, geared toward the specific needs of EID-CT. These algorithms need to be refined for PCD-CT due to their more complex data structure and the inherently available multi-energy data [3,20]. The QIR was designed to address these specific requirements.

This is the first study to systematically assess the use of QIR for UHR clinical PCD-CT imaging of the lung. While QIR-4 achieved the lowest noise level and the highest SNR of all reconstructions, scores from subjective analysis indicated that QIR-3 outperformed QIR-4 in terms of overall image quality and sharpness. Therein, QIR-3 slightly outperformed QIR-4 in terms of spatial resolution, as demonstrated with TTF analysis in the phantom. In general, TTF was slightly higher for low QIR levels than for high QIR levels, which is a well-known phenomenon from previous IR algorithms [25,32,33,34].

In this regard, it should be noted that while spatial resolution is an essential parameter in lung imaging, its impact on nodule detection may only be minor. Ichikawa et al. [35] demonstrated that the use of edge enhancement reconstruction kernels does not necessarily improve nodule visibility. Certainly, the effect of spatial resolution must be assessed in future PCD-CT studies. Additionally, in an upcoming software release the manufacturer intends to further refine QIR to maintain the objective spatial resolution even further. 

Our study demonstrated equal image noise texture among QIR-off and all QIR strength levels. Image noise texture may have an impact on perceived image quality and diagnostic confidence [21,36]. Previous IRs designed for EID-CT have been shown to change noise texture by altering the noise frequency distribution. Specifically, the change in image texture and appearance as seen with previous IRs was often caused by a shift of central noise frequency toward lower values [32,37,38,39,40]. In our NPS analysis, we demonstrated that average and peak noise frequency remained virtually identical among all reconstructions (maximum deviation of 6.7% for QIR-4), thus corroborating the fact that QIR does not suffer from the limitations of previous IRs. Consequently, QIR enables considerable noise reductions without compromising image texture.

Moreover, our results indicate that mean CT attenuation was comparable among reconstructions with clinically negligible (though statistically significant) absolute differences of a maximum of 4 HU between two reconstructions. Such stability is important for example, in automated lung emphysema imaging or nodule volumetry [41] in which attenuation thresholds are being used to distinguish healthy from affected lung parenchyma.

Lastly, we would like to address the radiation dose of our protocol. The average volume CT dose index (CTDI_vol_) in our patient cohort was 1 ± 0.6 mGy. A recent study assessing the radiation dose of low-dose lung cancer screening CT in 12,529 patients from 72 institutions found an average CTDI_vol_ of 2.4 ± 2 mGy. The American College of Radiology (ACR) recommends low dose scans to have CTDI_vol_ values of 3 mGy or less [42]. Thus, despite the very low radiation dose in our study cohort, the PCD-CT system enabled high quality lung imaging in all patients.

Our study had several limitations. First, all data was collected at a single healthcare center. Second, while in range of similar studies [24,28,43], the number of patients was limited. Futures studies encompassing a larger number of study subjects are desirable to confirm our results. Third, we did not assess the impact of QIR on diagnostic performance. Finally, we did not compare the performance of PCD-CT to EID-CT. This should be addressed in future phantom and clinical studies in order to establish the magnitude of the benefit of PCD-CT for lung imaging.

In conclusion, we recommend quantum iterative reconstruction at a strength level of 3 for low-dose, ultra-high-resolution photon-counting detector CT of the lung as it provides the best overall performance with regard to quantitative and qualitative image quality. Future studies should assess the impact of QIR on diagnostic accuracy and its potential for radiation dose reduction.

## Figures and Tables

**Table 1:** High-contrast TTF_50_ and TTF_10_ frequency shifts in percentage differences for QIR-1 to 4 levels in comparison with QIR-off.

Algorithm	TTF_50_	TTF_10_
QIR 1	0	2.7
QIR 2	−0.2	3.6
QIR 3	−1.8	2.1
QIR 4	−2.0	6.1

**Table 2:** **The** NPS peak frequency shifts in percentage differences for QIR levels from 1 to 4 in comparison with QIR-off.

Algorithm	NPS Peak Frequency Shifts (%)
QIR-1	0.0
QIR-2	0.0
QIR-3	0.0
QIR-4	−6.7

**Table 3:** Detailed overview of the results from quantitative and qualitative analysis in patients.

	QIR-Off	QIR-1	QIR-2	QIR-3	QIR-4
Global Noise Index [HU]	202 ± 34	178 ± 30	154 ± 26	130 ± 22	106 ± 18
Global SNR Index	4.4 ± 0.8	5 ± 0.9	5.7 ± 1.1	6.7 ± 1.3	8.2 ± 1.6
Mean Attenuation [HU]	−849 ± 53	−850 ± 53	−851 ± 52	−852 ± 52	−852 ± 52
Overall Image Quality	R1: 3; [3,4] R2: 3; [3,4]	R1: 4; [4,4] R2: 4; [4,4]	R1: 5; [4,5] R2: 5; [4,5]	R1: 5; [5,5] R2: 5; [5,5]	R1: 5; [4,5] R2: 5; [4,5]
Image Sharpness	R1: 4; [4,4] R2: 4; [3,4]	R1: 4; [4,5] R2: 4; [4,4.25]	R1: 5; [5,5] R2: 5; [5,5]	R1: 5; [5,5] R2: 5; [5,5]	R1: 4; [3,5] R2: 4; [4,5]
Image Noise	R1: 3; [3,3.25] R2: 3; [3,3]	R1: 4; [3,4] R2: 4; [3,4]	R1: 4; [4,5] R2: 4; [4,4]	R1: 5; [5,5] R2: 5; [5,5]	R1: 5; [5,5] R2: 5; [5,5]

Quantitative data is presented as mean ± standard deviation. Qualitative data is presented as median; (interquartile range) for reader 1 (R1) and reader 2 (R2), respectively.

## Data Availability

Upon reasonable request to the corresponding author.

## Abbreviations

PCD-CT: Photon-counting detector computed tomography; CNR: Contrast-to-noise ratio; EID-CT: Energy-integrating detector computed tomography; UHR: Ultra-high-resolution; IR: Iterative reconstruction; QIR: Quantum iterative reconstruction; TTF: Task transfer function; NPS: Noise power spectrum; BMI: Body mass index; CTDI_vol_: Volume CT dose index; DLP: Dose length product; SSDE: Size specific dose estimate; FBP: Filtered back projection; GNI: Global noise index; GSNRI: Global signal-to-noise ratio index; IQ: Image quality; SNR: Signal-to-noise ratio; SD: Standard deviation; ANOVA: Analysis of variance.
